# Rearfoot posture of *Australopithecus sediba* and the evolution of the hominin longitudinal arch

**DOI:** 10.1038/srep17677

**Published:** 2015-12-02

**Authors:** Thomas C. Prang

**Affiliations:** 1 Center for the Study of Human Origins, Department of Anthropology, New York University. 25 Waverly Place, New York, NY 10002; 2 New York Consortium in Evolutionary Primatology (NYCEP).

## Abstract

The longitudinal arch is one of the hallmarks of the human foot but its evolutionary history remains controversial due to the fragmentary nature of the fossil record. In modern humans, the presence of a longitudinal arch is reflected in the angular relationships among the major surfaces of the human talus and calcaneus complex, which is also known as the rearfoot. A complete talus and calcaneus of *Australopithecus sediba* provide the opportunity to evaluate rearfoot posture in an early hominin for the first time. Here I show that *A. sediba* is indistinguishable from extant African apes in the angular configuration of its rearfoot, which strongly suggests that it lacked a longitudinal arch. Inferences made from isolated fossils support the hypothesis that *Australopithecus afarensis* possessed an arched foot. However, tali attributed to temporally younger taxa like *Australopithecus africanus* and *Homo floresiensis* are more similar to those of *A. sediba*. The inferred absence of a longitudinal arch in *A. sediba* would be biomechanically consistent with prior suggestions of increased midtarsal mobility in this taxon. The morphological patterns in talus and calcaneus angular relationships among fossil hominins suggest that there was diversity in traits associated with the longitudinal arch in the Plio-Pleistocene.

The longitudinal arch of the human foot aids in propulsion and represents an energy-saving and shock-absorbing mechanism that is part of a suite of midfoot stabilizing morphologies associated with a commitment to terrestrial locomotion^1^,^2^,^3^. Great apes lack these specializations for terrestrial locomotion and instead have much more mobile feet that are more suited to vertical climbing and arboreality^4^,^5^. Whether or not early hominins possessed a modern human-like longitudinal arch is controversial, in large part because few of the relevant foot fossils have been recovered. Of the hominin species that do preserve the relevant morphology, *Ardipithecus ramidus* (4.4 Ma) possessed an abducted hallux, which precludes a modern human-like longitudinal arch^6^. Foot fossils attributed to *A. afarensis* are found at Afar Locality 333 at Hadar, Ethiopia^7^,^8^, although there is a talus and two phalanges associated with the A.L. 288–1 partial skeleton. The *A. afarensis* foot skeleton has a mix of some ape-like traits (e.g., long, curved phalanges), and several traits derived in the direction of modern humans (e.g., talocrural joint orthogonal to tibia, robust calcaneal tuber). Previous researchers have suggested that *A. afarensis* lacked a longitudinal arch based on prominent tuberosities on the lateral cuneiform (A.L. 333–79) and naviculars (A.L. 333–36, −47)^9^,^10^,^11^. However, *Australopithecus afarensis* (3.8–2.9 Ma) is now generally agreed to have had a longitudinal arch, as demonstrated most clearly in the morphology of a complete fourth metatarsal^2^,^8^ (but see ref. 12 for an alternative view) and the attribution of the Laetoli fossil footprints (3.7 Ma) to this taxon^13^,^14^.

The OH 8 fossils represent one of the most complete early hominin feet ever discovered^15^. Although originally assigned to *Homo habilis*, its taxonomic affinity is controversial given the co-occurrence of *H. habilis* and *Paranthropus boisei* at Olduvai Gorge *c.* 1.8 Ma^16^. A variety of conclusions have been drawn based on the morphology of OH 8, but the current consensus is that it is more similar to modern humans than to apes in ankle and midtarsal morphology, hallucal adduction, and metatarsal robusticity patterns^5^,^10^,^17^,^18^,^19^. There are some ape-like aspects of its trochlear morphology, but the talocrural joint is oriented orthogonally to the tibia, as it is in all bipedal hominins^5^. Whether or not OH 8 possessed a longitudinal arch is controversial and unresolved. Day and Wood^20^ showed that the talar declination angle of the OH 8 talus is ape-like, which would imply that this individual lacked a longitudinal arch. Furthermore, the metatarsal torsion pattern of OH 8 is ‘intermediate’ between modern humans and apes with a more medially oriented second metatarsal, which has been suggested to be related to longitudinal arch height^21^. Oxnard and Lisowski^22^ suggested that the transverse arch was re-constructed abnormally high in OH 8 and that a more anatomically accurate articulation of the tarsus results in a lower transverse arch, though this is not a uniquely modern human trait^23^,^24^,^25^. Whether *Paranthropus* (~2.7–1.2 Ma) or *Australopithecus africanus* (~2.5–2.0 Ma) possessed an arched foot has never been fully investigated, in part due to paucity of fossils and the difficulty in taxonomic assignation of foot fossils without craniodental material.

*Australopithecus sediba* (2.0 Ma) has been reported to have a longitudinal arch, despite the presence of ape-like features associated with greater midfoot mobility and a gracile calcaneal tuber^26^,^27^. The adult female individual of *Australopithecus sediba* (MH2), is represented by a talus, calcaneus, and distal tibia^26^,^28^. The calcaneus is ape-like in calcaneal tuber robusticity, its more dorsal orientation of its lateral plantar process (LPP), and mobile subtalar joint^26^,^27^. The talus is mosaic with a derived talocrural joint and a greatly enlarged talar head. However, it has been suggested that both the calcaneocuboid joint and the triceps surae attachment were plantarly oriented and thus human-like, indicating a dorsally elevated calcaneus consistent with the presence of a modern human-like longitudinal arch^26^,^27^. Furthermore, the MH2 calcaneus (U.W. 88–99) has a scar on the plantar surface consistent with an attachment for the long plantar ligament. A similar ligamentous scar can be seen on the 2.6 Ma calcaneus from the Omo Shungura Formation, Ethiopia (Omo 33-74-896)^19^ as well as the A.L. 333 (−55, −8) calcanei. However, hypotheses related to articular facet orientation have not been tested in extant hominoids or fossil hominins, including *A. sediba*.

The human foot has been described as a ‘twisted plate’^29^,^30^ wherein the rearfoot is in contact with the substrate in mild varus, the midfoot is elevated, and the forefoot is in contact with the substrate in pronation^30^. Although anatomists created this analogy to describe the human foot, it also applies to plantigrade apes. The African ape rearfoot is inverted and makes contact with the substrate during the stance phase of the terrestrial gait cycle, which is a posture that has been termed ‘inverted heel-strike plantigrady’, while the mid- and forefoot are everted^26^,^31^,^32^. The magnitude of the ‘twist’ in the plate is proportional to the degree of transverse tarsal joint supination-pronation, such that the foot becomes ‘untwisted’ when the forefoot is supinated and the rearfoot is in valgus^30^. Therefore, the pronation ‘twist’ of the midtarsus must occur in both apes and humans during closed chain terrestrial locomotion. Plantigrade apes possess modifications of the transverse tarsal joint relative to non-plantigrade taxa that reflect these differences, such as a more plantarly oriented talonavicular joint, and a tall calcaneocuboid joint relative to its width^31^. The bony morphology associated with the proximal portion of the human longitudinal arch is therefore rooted in an evolutionary history characterized by rearfoot plantigrady. The morphology of the rearfoot should further reflect modifications related to the dorsal elevation of the talus and calcaneus in the modern human foot associated with the evolution of the longitudinal arch.

The objective of this study is to test the hypothesis that the angular relationships among the major surfaces of the rearfoot reflect the presence of a longitudinal arch in humans. Morphologically, the medial longitudinal arch is composed of the talus, navicular, medial cuneiform and first metatarsal, whereas the lateral longitudinal arch is composed of the calcaneus, cuboid, and lateral metatarsals. The arched configuration of the modern human foot therefore results in the inclination of elements proximal to the talocrural axis of rotation (i.e., the calcaneus) and the declination of articulations distal to this axis (i.e., the talar head, metatarsals). In clinical contexts, longitudinal arch height in modern humans is typically measured using the angular inclination of the calcaneus or the position of the talonavicular joint relative to the substrate^33^,^34^,^35^. The talus and calcaneus represent both medial and lateral components of the longitudinal arch, and should be reliable indicators of longitudinal arch presence or absence (Fig. S1). First, if this hypothesis is true, the talonavicular and calcaneocuboid joints should be plantarly oriented relative to the ankle joint and the long axis of the calcaneus, respectively^20^,^36^,^37^, in order to maintain alignment with the transverse tarsal joint, which has a unique functional relationship with the longitudinal arch^4^,^38^,^39^. Second, to compensate for the elevation of the calcaneus, the angle of the subtalar joints (i.e., the anterior and posterior talocalcaneal joints) should increase relative to the long axis of the calcaneus concomitant with the more laterally positioned talus immediately above the calcaneus^23^. Third, the angulation of the attachment site for the triceps surae should decrease relative to the calcaneus to maintain the line of pull by the gastrocnemius and soleus muscles^26^, and to reduce the length of the moment arm of the triceps surae tendon, which has been shown to be a derived trait correlated with running economy in modern humans^40^. I then test the hypothesis that *Australopithecus sediba* possessed a longitudinal arch^26^,^27^. If *A. sediba* possessed a longitudinal arch, it should be more similar to humans than to non-human hominoids in its pattern of rearfoot angular relationships. These predictions are tested by calculating angles between the major surfaces of the talus and calcaneus in extant hominoids and fossil hominins.

## Results

**Figure 1:**
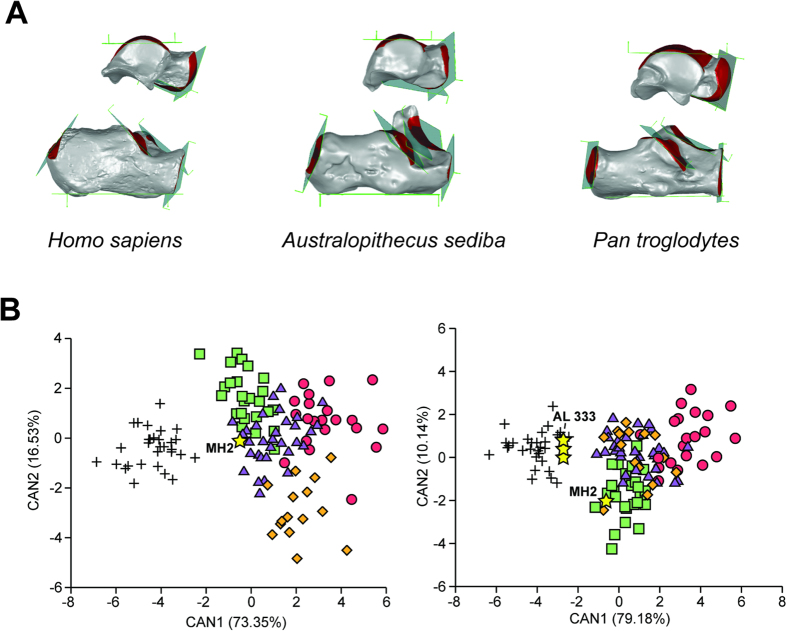
Rearfoot posture in humans, apes, and fossil hominins. (**A**) Angular relationships among the major surfaces of the hominoid talus and calcaneus. Note the dissimilarity between *A. sediba* and *H. sapiens* in talar head declination and other angular relationships. (**B**) Canonical Variates Analysis (CVA) of talus and calcaneus variables showing 90% of the total sample variance (left). CVA of reduced dataset of talus and calcaneus variables showing 89.3% of the total sample variance (right). *Homo* = plus, *Pan* = purple triangles, *Gorilla* = green squares, *Pongo* = orange diamonds, *Hylobates* = red circles. Humans are completely distinct from non-humans and *A. sediba* (MH2) is indistinguishable from an African ape, whereas *A. afarensis* is human-like.

**Figure 2:**
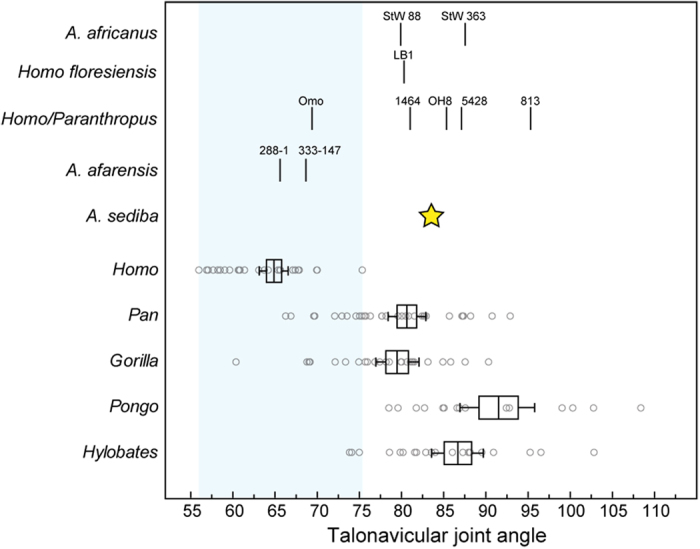
Talonavicular joint angle in extant hominoids and fossil hominins. Bars represent the mean, box represents plus/minus one standard error of the mean, whiskers represent the bootstrapped 95% confidence interval, and circles represent individual data points. Light blue bar represents the range of modern human variation. From smallest to largest values, *A. africanus* = StW 88, StW 363, *Homo/Paranthropus* = Omo 323-76-898, KNM-ER 1464, OH 8, KNM-ER 5428, KNM-ER 813, *A. afarensis* = A.L. 288–1, A.L. 333–147, *A. sediba* = U.W. 88–98. Note that all fossil hominins fall outside of the range of variation of modern humans except for *Australopithecus afarensis* specimens and Omo 323-76-898, which fall within the ranges of variation of *Homo*, *Pan*, and *Gorilla*.

A discriminant function analysis shows that seven angular measurements of the talus and calcaneus clearly differentiate modern humans from living apes and in a leave-one-out cross-validation modern humans are always correctly assigned (Fig. 1). Because not all angular measurements could be taken on all fossil specimens due to damage, separate analyses of subsets of the 7 angles were conducted to maximize inclusion of the fossils (Figures S3–S5, Tables S1–5). In general, angular relationships conform to predictions based on rearfoot posture in modern humans (Fig. 2, Table 1) with some notable exceptions. There is no difference among humans, gorillas, and orangutans in the angular orientation of the anterior talar facet of the calcaneus, which shows that it is probably a poor correlate of longitudinal arch presence (contra Morton, 1924). Additionally, there are some cases where other taxa approach modern humans in certain angular metrics such as the more plantarly oriented calcaneocuboid joint of gorillas compared to chimpanzees. However, no extant taxon has the full suite of traits that characterize the rearfoot of modern humans, especially the plantarly oriented talonavicular and calcaneocuboid joints, as well as the plantarly oriented triceps surae attachment. As such, subsequent analysis and discussion focuses on these variables when present in fossil hominins.

Confirming prior studies^2^,^8^,^14^, the geologically oldest taxon in the analysis, *A. afarensis*, is modern human-like in articular facet orientations and thus probably had a longitudinal arch. The two *A. afarensis* tali (A.L. 288–1, A.L. 333–147) are similar to modern humans in the plantar declination of the talar head relative to the ankle. The most complete *A. afarensis* calcaneus (A.L. 333–8) also has a modern human-like angular relationship between the triceps surae attachment and the posterior talocalcaneal joint relative to the long axis of the bone. A composite *A. afarensis* rearfoot is classified as *Homo sapiens* in a multivariate discriminant function analysis using a subset of the variables (Fig. 1). These results mirror a previous analysis of fourth metatarsal morphology in *A. afarensis*^2^, which showed that the long axis of the metatarsal shaft was oriented plantarly relative to the metatarsal base. The presence of a longitudinal arch in *A. afarensis* implies a commitment to terrestrial locomotion in this taxon^2^. Additional morphological evidence for terrestriality in *A. afarensis* derives from its adducted hallux^41^, the orthogonal orientation of its talocrural joint relative to the tibia^5^,^42^, its robust calcaneal tuber^43^,^44^, and its flattened posterior talocalcaneal joint^26^,^43^.

In contrast, none of the more recent fossil specimens analyzed here have similar angular morphologies to modern humans, and thus probably did not have a modern human-like longitudinal arch. The late occurring (2.0 Ma) *A. sediba*, which has an associated complete talus and calcaneus from the MH2 individual, lacks the plantarly oriented talonavicular and calcaneocuboid joints that are unique to modern humans, as well as the acute angle between the triceps surae attachment and the long axis of the calcaneus. All analyses clearly situate *A. sediba* among ape-like angular morphologies. The morphology of the *A. sediba* rearfoot is thus very similar to that of extant African apes. All fossil hominin tali attributed to temporally younger taxa like *A. africanus*, *H. habilis*, and *H. floresiensis* possess talonavicular joint angles outside of the range of variation for modern humans, except Omo 323-76-898 (Fig. 2). Although these fossil specimens (A.L. 288–1, A.L. 333–147, Omo 323-76-898) fall within the ranges of variation of humans and African apes, a multivariate analysis of 3 angular variables classifies *A. afarensis* as *Homo sapiens*, confirming its overall more human-like morphology, and all other hominin tali, including the Omo specimen, as non-human, which confirms its more ape-like talar head sagittal plane orientation (Fig. S5). Given that modern humans are a large-bodied, terrestrially adapted taxon, there is a possibility that some unique aspects of their talus and calcaneus articular orientation could be the result of allometry. Furthermore, the small size of many fossil hominins compared to most modern humans could result in the appearance of a low or absent longitudinal arch if there was a significant allometric component. To test for the effects of allometry, Pearson’s correlations were calculated between each variable and the overall size of the rearfoot calculated as the geometric mean of the square root of the talus and calcaneus surface areas (Table S5). There are no significant relationships between any angular variable and rearfoot size across each hominoid taxon. In *Gorilla gorilla*, there are two variables related to talar head morphology (troch-nav, cala-nav) which have a *p*-value just above the standard alpha of 0.05. However, the correlations (designated by the r values) are significantly less than 0.5, which indicates that it is probably not biologically meaningful (following ref. 45). Intraspecific rearfoot size does not explain the observed pattern of rearfoot angular relationships across hominoid primates. All fossil hominin tali from later than 2.5 Ma sampled here lack the derived rearfoot configuration characteristic of both modern humans and *A. afarensis* that has been associated with the presence of a longitudinal arch (Fig. S2).

## Discussion

The hard and soft tissue specializations of the modern human mid- and forefoot enable it to be mobile enough to conform to substrates during midstance and rigid enough to act as a propulsive lever during the toe-off stage of the gait cycle. These bony traits include the dorsoplantar expansion and concomitant flattening of the lateral tarsometatarsal joints^2^,^46^,^47^, the proximomedial positioning of the cuboid beak, the high medial torsion of the talar head, and the permanent adduction of the hallux. Great apes lack these bony features and instead have much more mobile tarsometatarsal, calcaneocuboid, and talonavicular joints^4^,^46^,^48^,^49^. Among the soft tissue manifestations of this specialization is a well-developed plantar aponeurosis, which is an important component of the midfoot stabilizing ‘windlass mechanism’^2^,^50^. Since the plantar aponeurosis attaches distally to the bases of all five proximal pedal phalanges, passive dorsiflexion of the metatarsophalangeal joints results in the tautening of the plantar soft tissues, which mildly flexes the metatarsus and raises the longitudinal arch as the rearfoot supinates in response to the action of the triceps surae^2^,^50^,^51^. Thus, the height of a modern human individual’s longitudinal arch changes throughout the stance phase of the gait cycle. Recent biomechanical analyses have shown that modern humans with a lower longitudinal arch tend to have greater lateral midfoot mobility^52^, greater midfoot pronation, and increased dorsiflexion at the hallucal metatarsophalangeal joint^53^,^54^.

The shape of the tarsometatarsal joints has been shown to be a reliable osteological correlate of such tarsometatarsal mobility in modern humans and great apes^46^,^54^. The Malapa Hominin 1 (MH1) individual of *A. sediba* has the most convex fourth metatarsal of any australopith yet discovered and its curvature value falls outside of the range of modern human variation^27^, implying that this individual may have possessed a ‘midtarsal break’^54^. While it is certainly possible that the MH1 fourth metatarsal is representative of the extreme upper end of variation for the *A. sediba* species and thus not meaningfully dissimilar from modern humans, the remaining pedal elements (as represented by MH2) show that *A. sediba* was distinct from humans and some other fossil hominins, like *A. afarensis*. The distributions of values for single metrics often overlap among species with different adaptations (e.g., humans and chimpanzees), but in most cases these traits probably do not covary with other functionally relevant traits in the same manner across taxa, and thus probably do not exist within similar morphological and, by extension, functional systems. No modern humans possess the totality of features that characterize the foot of *A. sediba*. If form follows function, this observation militates against modern human-like foot function in *A. sediba*, even when considering the significant range of variation in single traits in modern humans since functional systems, and the evolutionary processes that produce and maintain them, are intrinsically multivariate^55^. The evolution of these morphologies probably involved selection on the total function system comprised of the foot and lower limb in hominins, rather than micro anatomical regions given that morphological structures evolve in a correlated fashion^56^,^57^,^58^,^59^. Although there is considerable variation within species and metric overlap between them, the geometric configuration of talus and calcaneus articular surfaces in *A. sediba* is consistent with suggestions of increased midtarsal mobility^26^,^27^,^54^ and the absence of a longitudinal arch.

Paleoecological data combined with morphological data on the postcranium of *Australopithecus* suggest a possible paleobiological division between *A. sediba* and other hominins. Recent analyses of stable isotopes and dental microwear showed that the diet of *A. sediba* was most similar to chimpanzees and *Ardipithecus ramidus* in their preferential consumption of C_3_ foods in the presence of abundant C_4_ foods^60^. Other hominins like *A. afarensis* and *A. africanus* may have been more mixed in their C_3_/C_4_ consumption. *Australopithecus anamensis*, the likely precursor to *A. afarensis*, is not as well-known postcranially, but at 4.1 Ma it apparently possessed a modern human-like distal tibia morphology that is more derived than *A. sediba*^26^. The *A. anamensis*-*A. afarensis* lineage might have become adapted for terrestriality, with taxa in South Africa never developing the traits seen in east African *Australopithecus* such as a robust calcaneal tuber^26^,^44^, human like limb size proportions^61^, and a longitudinal arch. The phylogenetic relationships among Plio-Pleistocene hominins are unresolved but most analyses support *A. afarensis* nearer the base of the hominin clade and *A. africanus* as more closely related to *Homo*^62^,^63^. If *A. afarensis* contributed to the ancestry of *Homo*^64^, there would need to be an evolutionary reversal in morphologies related to the longitudinal arch, as is the case for other areas of anatomy^26^,^44^,^61^, with no specific functional explanation. If not, the presence of a longitudinal arch in *A. afarensis* and modern humans would have necessarily evolved independently. This hypothesis is supported by the ape-like talonavicular joint angle in the LB1 individual of *Homo floresiensis* and purported *Homo* fossils (e.g., OH 8, KNM-ER 813, KNM-ER 5428). *Australopithecus sediba* was originally suggested to represent a probable ancestral condition for *Homo*^28^, but recent analyses suggest that it is no more closely related to *Homo* than to *A. africanus*^63^ (but see ref. 65), whose own phylogenetic position has been unstable^66^. The alternative evolutionary hypothesis is that *A. afarensis* is ancestral to *Homo* and the derived craniodental traits shared by *A. africanus*, *A. sediba*, and *Homo* evolved via homoplasy. This would require at least one evolutionary reversal to explain the ape-like morphologies in *H. floresiensis* and other purported *Homo* fossils. Regardless of which phylogenetic hypothesis is adopted, homoplasy must have played a role in the evolution of morphologies associated with the longitudinal arch—either in the convergence of *A. afarensis* with modern humans or through evolutionary reversals in later hominins like *A. sediba*, *A. africanus*, and *Homo*. Phylogenetic analyses that consider postcranial morphology have the potential to resolve these issues and more research in this area is sorely needed.

## Conclusion

The pattern of rearfoot angular relationships in extant taxa suggests that *A. sediba* lacked a longitudinal arch, unlike the more derived *A. afarensis*. The inferred absence of an arch in *A. sediba* is consistent with suggestions of increased midtarsal mobility^26^,^27^. The two major traits that distinguish the modern human foot from the ape foot are the longitudinal arch and the adducted hallux^36^. One of the emerging hypotheses in paleoanthropology is that early hominins were diverse in their locomotor adaptation as evidenced by morphological diversity in postcranial traits related to arboreality and terrestriality^10^,^11^,^67^. Recently discovered fossils from Woranso Mille, Ethiopia suggest that at *c.* 3.4–3.3 Ma there existed two pedal morphs in East Africa—one with a more modern human-like degree of hallucal adduction and the other with a more *Ardipithecus*-like hallux^68^. Whether the Burtele hominin is a late-surviving member of *Ardipithecus ramidus*, or belongs to *Australopithecus anamensis* or *Australopithecus deyiremeda*^69^ is unknown. The data presented here suggest that there was also diversity among hominins in traits associated with the longitudinal arch. If the derived traits in the *A. afarensis* foot are convergences, the evolution of the longitudinal arch in the *Homo* lineage probably occurred *c.* 2.0 Ma, perhaps as part of a shift to a postcranial body plan adapted for the type of exclusive terrestrial bipedalism seen in *Homo erectus*^70^,^71^. It is difficult to incorporate phylogenetic uncertainty into empirical reconstructions of evolutionary patterns, especially in cases of evolutionary singularities such as hominin bipedalism. However, the currently available paleontological and neontological data do not support scenarios invoking repeated evolutionary reversals in traits associated with the longitudinal arch, and other areas of anatomy^26^,^44^,^61^, especially without adaptive explanation. These hypotheses could be further evaluated with additional discoveries and analyses of *Australopithecus* and *Homo* foot fossils.

## Methods

### Extant and fossil sample

The extant sample derives from the American Museum of Natural History (AMNH), United States National Museum (USNM), Cleveland Museum of Natural History (CMNH), Academy of Natural Sciences Philadelphia (ANSP), and collections at New York University (NYU), and SUNY Stony Brook. Original fossil material from Sterkfontein and Malapa was examined at the University of the Witwatersrand in Johannesburg, South Africa. The *Australopithecus sediba* talus (U.W. 88–98) and calcaneus (U.W. 88–99) fossils were originally CT scanned and digitally separated from one another with a previously published protocol^16^,^18^. All other observations of fossil hominin tali and calcanei were conducted using high quality casts housed at the Cleveland Museum of Natural History and the Center for the Study of Human Origins (CSHO) at New York University. There are several *Australopithecus* or *Homo/Paranthropus* tali that are well-known but too fragmentary to include in this study: AL 333–75 (talar head only), KNM-ER 1476 (lacks dorsal portion of talar head), StW 347 (lacks posterior half of trochlea), StW 486 (lacks posteromedial corner of talar trochlea), StW 102 (lacks talar head), SKX 42695 (lacks talar head), TM 1517 (lacks plantar two-thirds of talar head and posterolateral corner of trochlea).

### Data acquisition

Bones were scanned with a NextEngine desktop laser scanner in at least two orientations with ten rotations per scan. A three-dimensional surface mesh was created for each bone by combining the scans of both orientations in Geomagic Studio software and cleaning imperfections (e.g., filling holes). Articular surfaces were segmented from non-articular surfaces using Geomagic Studio software (following refs 72, 73). Although non-articular, the triceps surae attachment site on the proximal end of the calcaneal tuber was also segmented from the bone since this surface is included by previous researchers in longitudinal arch hypotheses^26^,^27^ and since it is directly related to the function of the triceps surae in taxa that differ in heel elevation (e.g., those with a longitudinal arch). Three-dimensional angles between surfaces were quantified by calculating the inverse cosine of the dot product between normal vectors defined by least squares planes fit to each surface (following refs 72, 73). Calcaneus angles were calculated relative to a basal plane that is parallel to the long axis of the bone defined by three non-collinear landmarks: most plantar point of the calcaneocuboid facet, most plantar point of the medial plantar process, and most plantar point of the lateral plantar process or the peroneal trochlea (whichever is more plantarly oriented). Thus, the basal plane of the calcaneus is homologous across all specimens regardless of the presence or absence of the lateral plantar process (e.g., in humans and *Australopithecus afarensis*). These angles were specifically chosen to reflect the orientation of (1) the transverse tarsal joint (base-cub, troch-nav, troch-cala, nav-cala), (2) the subtalar joint (base-tala, base-talp), and (3) the triceps surae attachment (base-ts) in accordance with the predictions outlined above.

### Statistical analyses

Multivariate canonical variates (CVA) and discriminant function (DFA) analyses were conducted using 7 variables: troch-nav angle, troch-cala angle, nav-cala angle, calcaneocuboid angle, talp angle, tala angle, and triceps surae angle. Several subsets of multivariate analyses were conducted in order to maximize inclusion of various fossil specimens. Fossil hominins were added ‘*a posteriori’* as unknown specimens to be placed among extant hominoids. Pearson’s correlations were used to test for the effects of allometry (following ref. 28) on talus and calcaneus angular variables. A geometric mean of the square roots of the total bony surface area of the talus and calcaneus was used as a proxy for overall rearfoot size. All statistical analyses were conducted using PAST^74^,^75^.

## Additional Information

**How to cite this article**: Prang, T.C. Rearfoot posture of *Australopithecus sediba* and the evolution of the hominin longitudinal arch. *Sci. Rep.* **5**, 17677; doi: 10.1038/srep17677 (2015).

## Supplementary Material

Supplementary Information

## Figures and Tables

**Table 1:** Summary statistics for talus and calcaneus angles.

Taxon	Talus			Calcaneus			
	troch-nav	troch-cala	cala-nav	base-talp	base-tala	base-ts	base-cub
*Homo sapiens*	65 ± 5	29 ± 2	88 ± 6	44 ± 4	43 ± 6	62 ± 7	106 ± 4
N = 30	(58, 77)	(12, 43)	(77, 98)	(34, 51)	(31, 56)	(45, 75)	(97, 114)
*Pan troglodytes*	81 ± 7	16 ± 6	89 ± 4	35 ± 6	25 ± 7	79 ± 6	91 ± 4
N = 33	(68, 94)	(6, 31)	(78, 100)	(26, 45)	(11, 43)	(67, 92)	(84, 100)
*Gorilla gorilla*	80 ± 7	16 ± 6	94 ± 7	45 ± 5	40 ± 8	81 ± 5	100 ± 5
N = 25	(62, 92)	(4, 28)	(84, 105)	(35, 59)	(25, 56)	(72, 89)	(92, 110)
*Pongo pygmaeus*	92 ± 9	16 ± 4	81 ± 9	37 ± 12	40 ± 12	75 ± 6	83 ± 4
N = 15	(81, 110)	(10, 22)	(60, 93)	(15, 57)	(21, 65)	(67, 87)	(75, 92)
*Hylobates*	87 ± 7	16 ± 6	81 ± 7	30 ± 7	24 ± 10	96 ± 10	88 ± 6
N = 21	(76, 104)	(6, 29)	(62, 92)	(14, 42)	(8, 44)	(77, 122)	(77, 99)
A.L. 288–1	67	29	92	—	—	—	—
A.L. 333–147	69	30	85	—	—	—	—
A.L. 333–8	—	—	—	46	26	65	—
MH2	84	22	89	49	51	74	98
StW 88	80	19	87	—	—	—	—
StW 363	88	30	73	—	—	—	—
LB1	80	19	80	—	—	—	—
OH 8	85	10	86	47	34	—	108
Omo 323-76-898	69	25	100	—	—	—	—
Omo 33-74-896	—	—	—	43	45	75	99
KNM-ER 813	95	15	74	—	—	—	—
KNM-ER 1464	81	22	83	—	—	—	—
KNM-ER 5428	87	—	—	—	—	—	—

Mean ± SD with minimum and maximum values. troch = talar trochlea, nav = talus navicular facet, cala = talus anterior calcaneal facet, talp = calcaneus posterior talar facet, tala = calcaneus anterior talar facet, ts = calcaneus triceps surae attachment, cub = calcaneus cuboid facet. StW 88, StW 363 = *A. africanus*, A.L. 288–1, A.L. 333–147, A.L. 333–8 = *A. afarensis*, OH 8, KNM-ER 5428, KNM-ER 1464, KNM-ER 813, Omo 323-76-898, and Omo 33-74-896 = *Homo/Paranthropus*, MH2 (U.W. 88–98/99) = *A. sediba*.
