# Supplementary material for: Rearfoot posture of Australopithecus sediba and the evolution of the hominin longitudinal arch
Source: Sci Rep. 2015 Dec 2;5:17677. doi: 10.1038/srep17677 (PMC4667273; doi:10.1038/srep17677)
Supplement: Supplementary Information [file srep17677-s1.doc]

**Supplementary Information (SI)**

**Rearfoot posture of *Australopithecus sediba* and the evolution of the hominin longitudinal arch**

Thomas Cody Prang^1,2*^

^1^Center for the Study of Human Origins, Department of Anthropology, New York University. 25 Waverly Place, New York, NY 10002.

^2^New York Consortium in Evolutionary Primatology (NYCEP)

| Table S1. Canonical Variates Analysis | | | | |
| --- | --- | --- | --- | --- |
| Percent | 73.35 | 16.53 | 7.49 | 2.63 |
| Eigenvalue | 7.683 | 1.731 | 0.784 | 0.275 |
|  | CAN1 | CAN2 | CAN3 | CAN4 |
| troch-nav | 0.0172 | -0.0035 | -0.0112 | -0.0129 |
| troch-cala | -0.0408 | -0.0246 | 0.0031 | 0.0141 |
| cala-nav | -0.0021 | 0.0118 | -0.0033 | -0.0123 |
| base-talp | -0.0161 | 0.0129 | -0.0423 | -0.0041 |
| base-tala | -0.0236 | -0.0105 | -0.1036 | 0.0396 |
| base-ts | 0.0193 | 0.0179 | 0.0046 | 0.0221 |
| base-cub | -0.0123 | 0.0095 | -0.0063 | 0.0027 |

| Table S2. Discriminant Function Analysis | | | | | | | | | | | | |
| --- | --- | --- | --- | --- | --- | --- | --- | --- | --- | --- | --- | --- |
| Resubstitution (95.6% correct) | | | | | |  | Cross-validation (89.4% correct) | | | | | |
|  | *Homo* | *Pan* | *Gorilla* | *Pongo* | *Hylobates* |  |  | *Homo* | *Pan* | *Gorilla* | *Pongo* | *Hylobates* |
| *Homo* | **30** | 0 | 0 | 0 | 0 |  | *Homo* | **30** | 0 | 0 | 0 | 0 |
| % | **100.0** | 0.0 | 0.0 | 0.0 | 0.0 |  | % | **100.0** | 0.0 | 0.0 | 0.0 | 0.0 |
|  |  |  |  |  |  |  |  |  |  |  |  |  |
| *Pan* | 0 | **29** | 2 | 1 | 1 |  | *Pan* | 0 | **28** | 3 | 1 | 1 |
| % | 0.0 | **88.0** | 6.0 | 3.0 | 3.0 |  | % | 0.0 | **85.0** | 9.0 | 3.0 | 3.0 |
|  |  |  |  |  |  |  |  |  |  |  |  |  |
| *Gorilla* | 0 | 1 | **24** | 0 | 0 |  | *Gorilla* | 0 | 3 | **22** | 0 | 0 |
| % | 0.0 | 4.0 | **96.0** | 0.0 | 0.0 |  | % | 0.0 | 12.0 | **88.0** | 0.0 | 0.0 |
|  |  |  |  |  |  |  |  |  |  |  |  |  |
| *Pongo* | 0 | 2 | 0 | **13** | 0 |  | *Pongo* | 0 | 2 | 0 | **12** | 1 |
| % | 0.0 | 13.0 | 0.0 | **87.0** | .0 |  | % | 0.0 | 13.0 | 0.0 | **80.0** | 7.0 |
|  |  |  |  |  |  |  |  |  |  |  |  |  |
| *Hylobates* | 0 | 3 | 0 | 1 | **17** |  | *Hylobates* | 0 | 4 | 0 | 2 | **15** |
| % | 0.0 | 14.0 | 0.0 | 5.0 | **81.0** |  | % | 0.0 | 19.0 | 0.0 | 10.0 | **71.0** |

| Table S3. Canonical Variates Analysis 2 | | | | |
| --- | --- | --- | --- | --- |
| Percent | 79.18 | 10.14 | 7.48 | 3.192 |
| Eigenvalue | 6.479 | 0.831 | 0.612 | 0.261 |
|  | CAN1 | CAN2 | CAN3 | CAN4 |
| troch-nav | 0.0182 | -0.0165 | -0.0168 | 0.0102 |
| troch-cala | -0.0481 | -0.0055 | 0.0034 | -0.0135 |
| cala-nav | -0.0006 | 0.0192 | 0.0016 | 0.0144 |
| base-talp | -0.0158 | 0.0386 | -0.0331 | 0.0068 |
| base-tala | -0.0276 | 0.0224 | -0.1001 | -0.0413 |
| base-ts | 0.0236 | 0.0118 | 0.0075 | -0.0213 |
| Reduced dataset for inclusion of *A. afarensis* | | | | |

| Table S4. Discriminant Function Analysis 2 | | | | | | | | | | | | |
| --- | --- | --- | --- | --- | --- | --- | --- | --- | --- | --- | --- | --- |
| Resubstitution (84.68% correct) | | | | | |  | Cross-validation (82.26% correct) | | | | | |
|  | *Homo* | *Pan* | *Gorilla* | *Pongo* | *Hylobates* |  |  | *Homo* | *Pan* | *Gorilla* | *Pongo* | *Hylobates* |
| *Homo* | **30** | 0 | 0 | 0 | 0 |  | *Homo* | **30** | 0 | 0 | 0 | 0 |
| % | **100.0** | 0.0 | 0.0 | 0.0 | 0.0 |  | % | **100.0** | 0.0 | 0.0 | 0.0 | 0.0 |
|  |  |  |  |  |  |  |  |  |  |  |  |  |
| *Pan* | 0 | **28** | 1 | 3 | 1 |  | *Pan* | 0 | **28** | 1 | 3 | 1 |
| % | 0.0 | **85.0** | 3.0 | 9.0 | 3.0 |  | % | 0.0 | **85.0** | 3.0 | 9.0 | 3.0 |
|  |  |  |  |  |  |  |  |  |  |  |  |  |
| *Gorilla* | 0 | 2 | **21** | 2 | 0 |  | *Gorilla* | 0 | 3 | **20** | 2 | 0 |
| % | 0.0 | 8.0 | **84.0** | 8.0 | 0.0 |  | % | 0.0 | 12.0 | **80.0** | 8.0 | 0.0 |
|  |  |  |  |  |  |  |  |  |  |  |  |  |
| *Pongo* | 0 | 3 | 2 | **10** | 0 |  | *Pongo* | 0 | 4 | 2 | **9** | 0 |
| % | 0.0 | 20.0 | 13.0 | **67.0** | 0.0 |  | % | 0.0 | 27.0 | 13.0 | **60.0** | 0.0 |
|  |  |  |  |  |  |  |  |  |  |  |  |  |
| *Hylobates* | 0 | 3 | 1 | 1 | **16** |  | *Hylobates* | 0 | 4 | 1 | 1 | **15** |
| % | 0.0 | 14.0 | 5.0 | 5.0 | **76.0** |  | % | 0.0 | 19.0 | 5.0 | 5.0 | **71.0** |
| Reduced dataset for inclusion of *A. afarensis* | | | | | | | | | | | | |

| Table S5. Pearson's correlations between geometric mean and each variable | | | | | | | | | | | | | | | |
| --- | --- | --- | --- | --- | --- | --- | --- | --- | --- | --- | --- | --- | --- | --- | --- |
|  |  | *Homo sapiens* | |  | *Pan troglodytes* | |  | *Gorilla gorilla* | |  | *Pongo pygmaeus* | |  | *Hylobates* | |
| Variable |  | r | *p* |  | r | *p* |  | r | *p* |  | r | *p* |  | r | *p* |
| troch-nav |  | -0.1243 | 0.5129 |  | -0.1736 | 0.3339 |  | -0.3912 | 0.0532 |  | -0.1292 | 0.6598 |  | -0.4233 | 0.1945 |
| troch-cala |  | 0.0123 | 0.9485 |  | -0.1362 | 0.4498 |  | 0.2978 | 0.1482 |  | -0.2317 | 0.4254 |  | 0.0075 | 0.9826 |
| cala-nav |  | 0.1345 | 0.4786 |  | 0.1396 | 0.4385 |  | 0.3773 | 0.0631 |  | 0.0355 | 0.9041 |  | 0.1894 | 0.5768 |
| base-talp |  | -0.0381 | 0.8414 |  | -0.0813 | 0.6529 |  | -0.1905 | 0.3616 |  | -0.3038 | 0.2911 |  | 0.1524 | 0.6546 |
| base-tala |  | -0.0028 | 0.9883 |  | -0.2293 | 0.1993 |  | -0.0305 | 0.8848 |  | -0.2661 | 0.3577 |  | 0.3715 | 0.2607 |
| base-ts |  | 0.1731 | 0.3604 |  | -0.0462 | 0.7984 |  | -0.3028 | 0.1412 |  | -0.4979 | 0.0699 |  | 0.1006 | 0.7686 |
| base-cub |  | 0.0945 | 0.6193 |  | 0.1486 | 0.4091 |  | 0.3175 | 0.1219 |  | -0.2307 | 0.4274 |  | 0.4534 | 0.1614 |


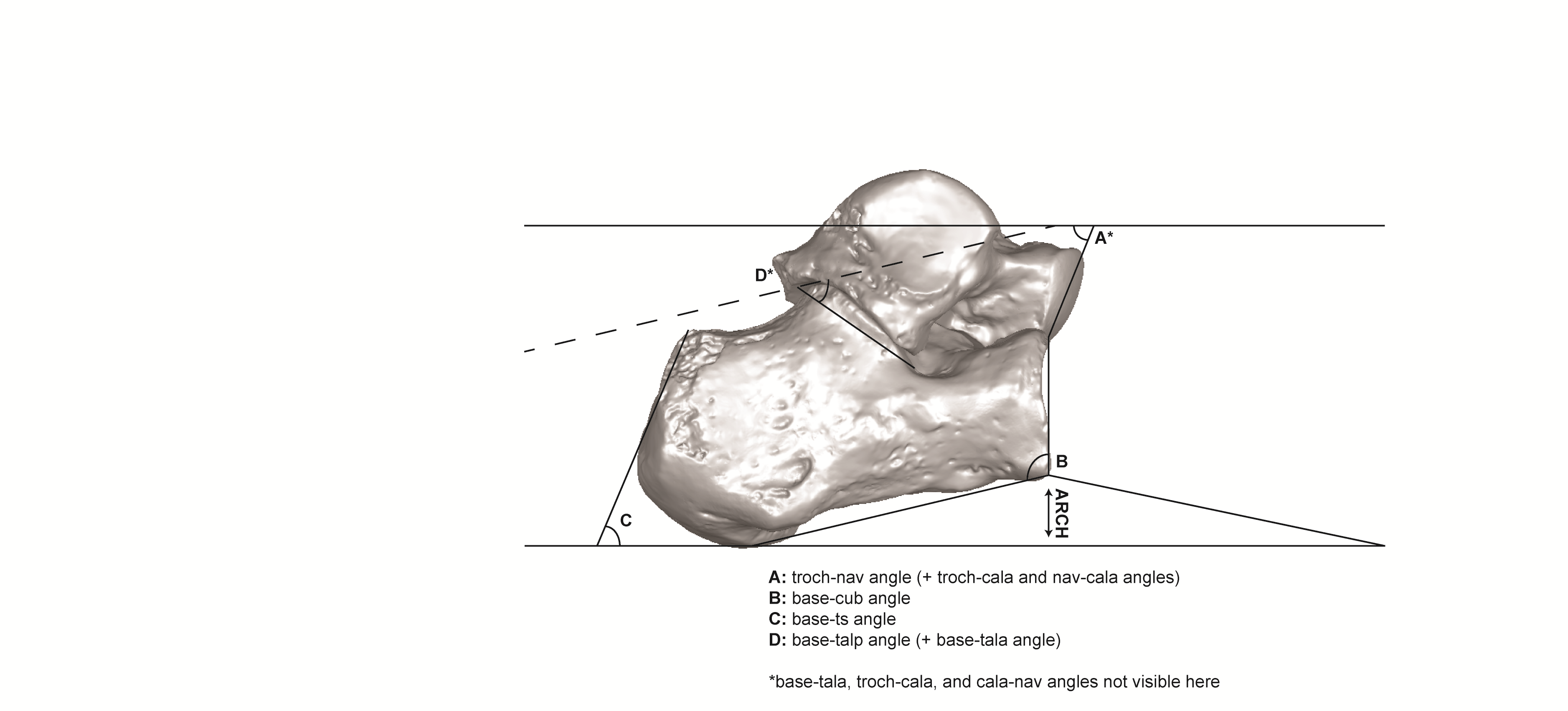


**Figure S1.** Geometry of the rearfoot hypothetically associated with the longitudinal arch. The troch-cala, nav-cala, and base-tala angles are obscured due to the digital articulation of these elements, but their predictions follow those for the troch-nav and base-talp angles (i.e., base-tala angle should be larger in taxa with a more distally elevated calcaneus, like base-talp; troch-cala should be larger in taxa with a more plantarly oriented talar head). The angle between the navicular facet (nav) and the anterior calcaneal facet (cala) is not specifically linked to longitudinal arch hypotheses, but given that fossil hominins may be autapomorphic relative to extant taxa, there is a possibility that a hominin with a high (i.e., ape-like) troch-nav angle, could compensate with a high (i.e., human-like) troch-cala angle. If so, this should result in a relatively smaller nav-cala angle. The dashed line is parallel to the basal plane of the calcaneus and the more dorsal line near the talus is parallel to the substrate plane beneath the calcaneus. Note that this study used a three-dimensional methodology for calculating angles between surfaces rather than a two-dimensional one, which is represented here. Therefore, this figure is for illustrative purposes only.


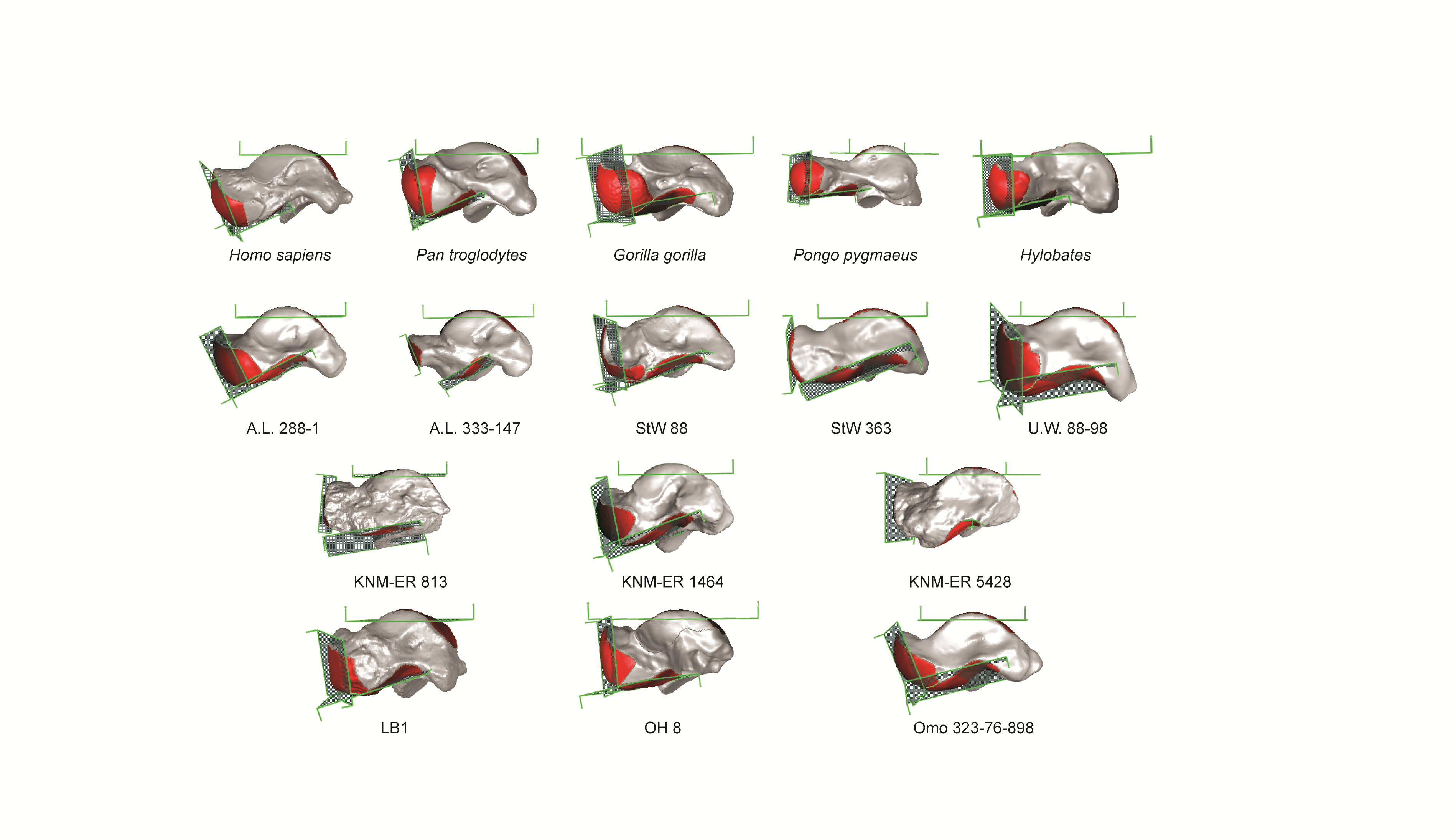


**Figure S2.** Talar articular angles in extant hominoids and fossil hominins. Note the plantarly oriented talar head of humans, followed by African apes (*Pan* and *Gorilla*), then by Asian apes (*Pongo* and *Hylobates*). The placement of the talus on the dorsal surface of the calcaneus, combined with the supinated rearfoot and the pronated forefoot (i.e., the twisted plate), necessitates that the talonavicular joint is plantarly oriented in African apes relative to Asian apes, which lack a terrestrial plantigrade rearfoot adaptation (Gebo, 1992). Humans take this African ape pattern to an extreme with the evolution of the longitudinal arch. Note the human-like morphology of *A. afarensis* (A.L. 288-1 and A.L. 333-147) and the distinctly gorilla-like morphology of *A. sediba* (U.W. 88-98). Most of the fossil hominins lack a plantarly oriented talar head associated with the longitudinal arch and instead have a distally facing navicular facet.


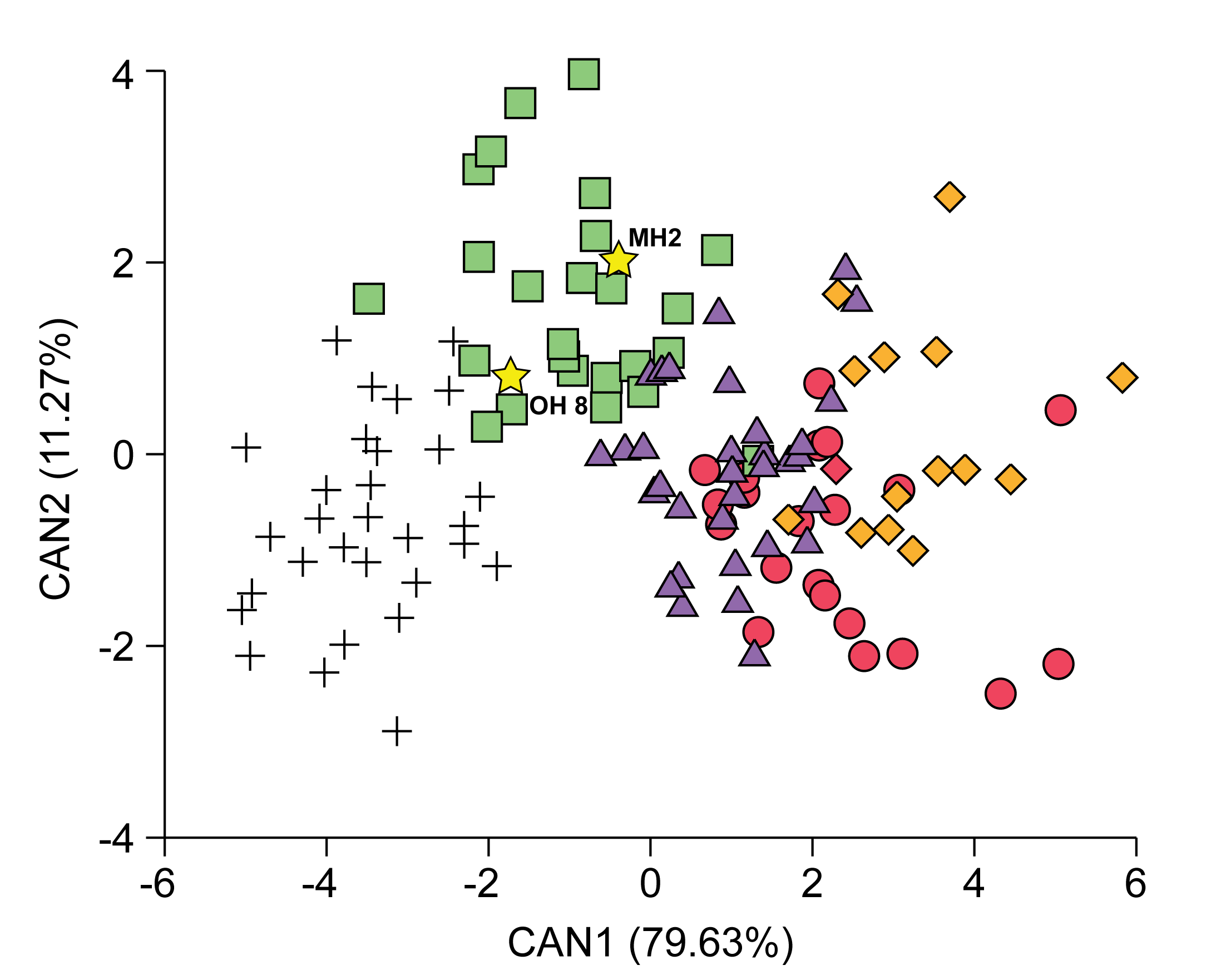


**Figure S3**. Canonical Variates Analysis (CVA) of 7 talus and calcaneus variables (troch-nav, troch-cala, cala-nav, base-talp, base-tala, base-cub) showing 90.9% of the total sample variance. The associated discriminant function analysis (DFA) classifies 93% of *Homo*, 76% of *Pan*, 88% of *Gorilla*, 67% of *Pongo*, and 52% of *Hylobates* correctly using cross-validation. *Homo* = plus, *Pan* = purple triangles, *Gorilla* = green squares, *Pongo* = orange diamonds, *Hylobates* = red circles. MH2 and OH 8 are both classified as *Gorilla* when added as ‘unknown’ or as *Homo sapiens*.


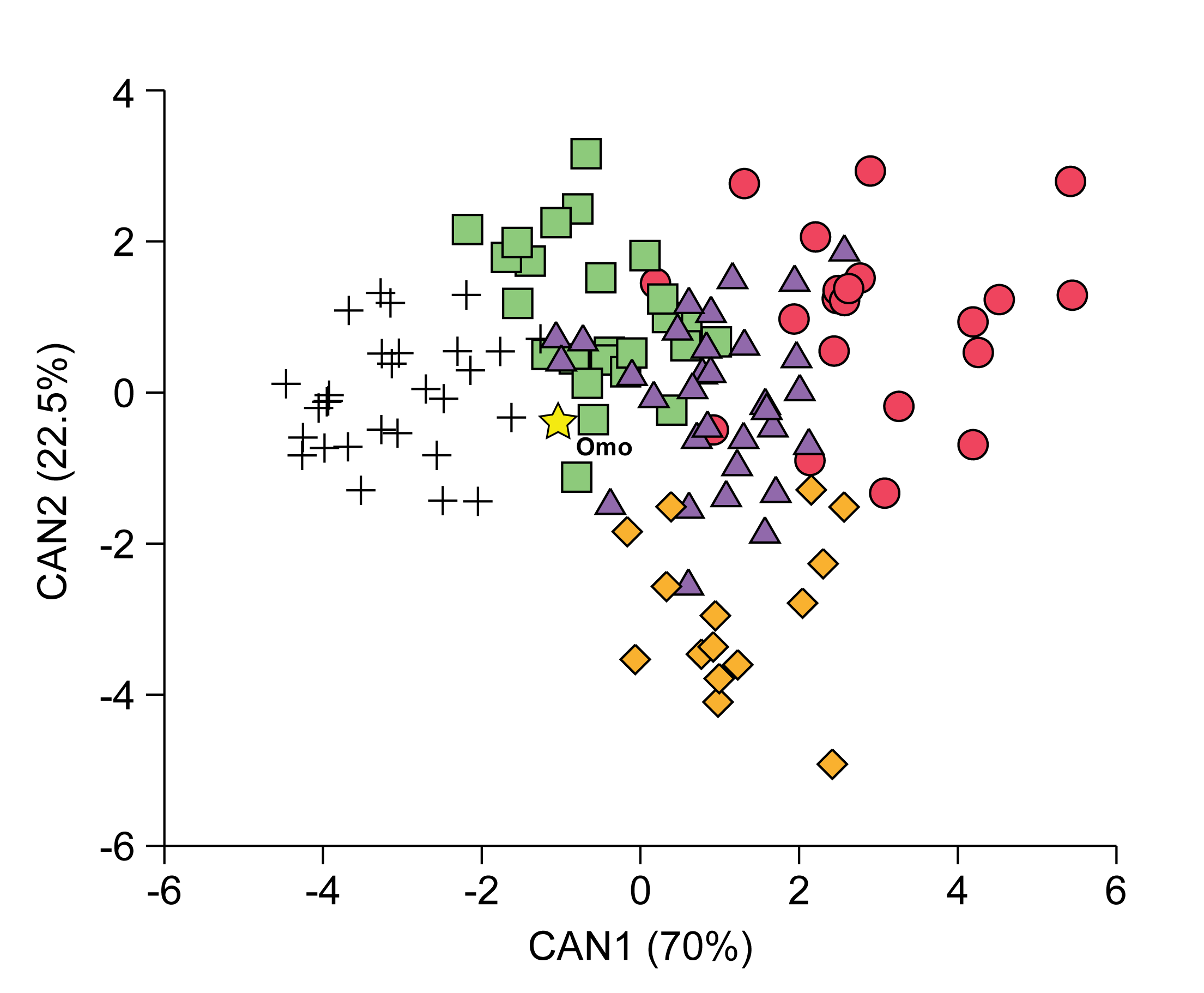


**Figure S4.** Canonical Variates Analysis (CVA) using 4 calcaneus variables (base-talp, base-tala, base-ts, base-cub), which shows 92.5% of the total sample variance. The associated discriminant function analysis (DFA) classifies 97% of *Homo*, 70% of *Pan*, 84% of *Gorilla*, 87% of *Pongo*, and 76% of *Hylobates* correctly using cross-validation. *Homo* = plus, *Pan* = purple triangles, *Gorilla* = green squares, *Pongo* = orange diamonds, *Hylobates* = red circles. MH2 and Omo 33-74-896 are both classified as *Gorilla* when added as ‘unknown’ or *Homo sapiens*.


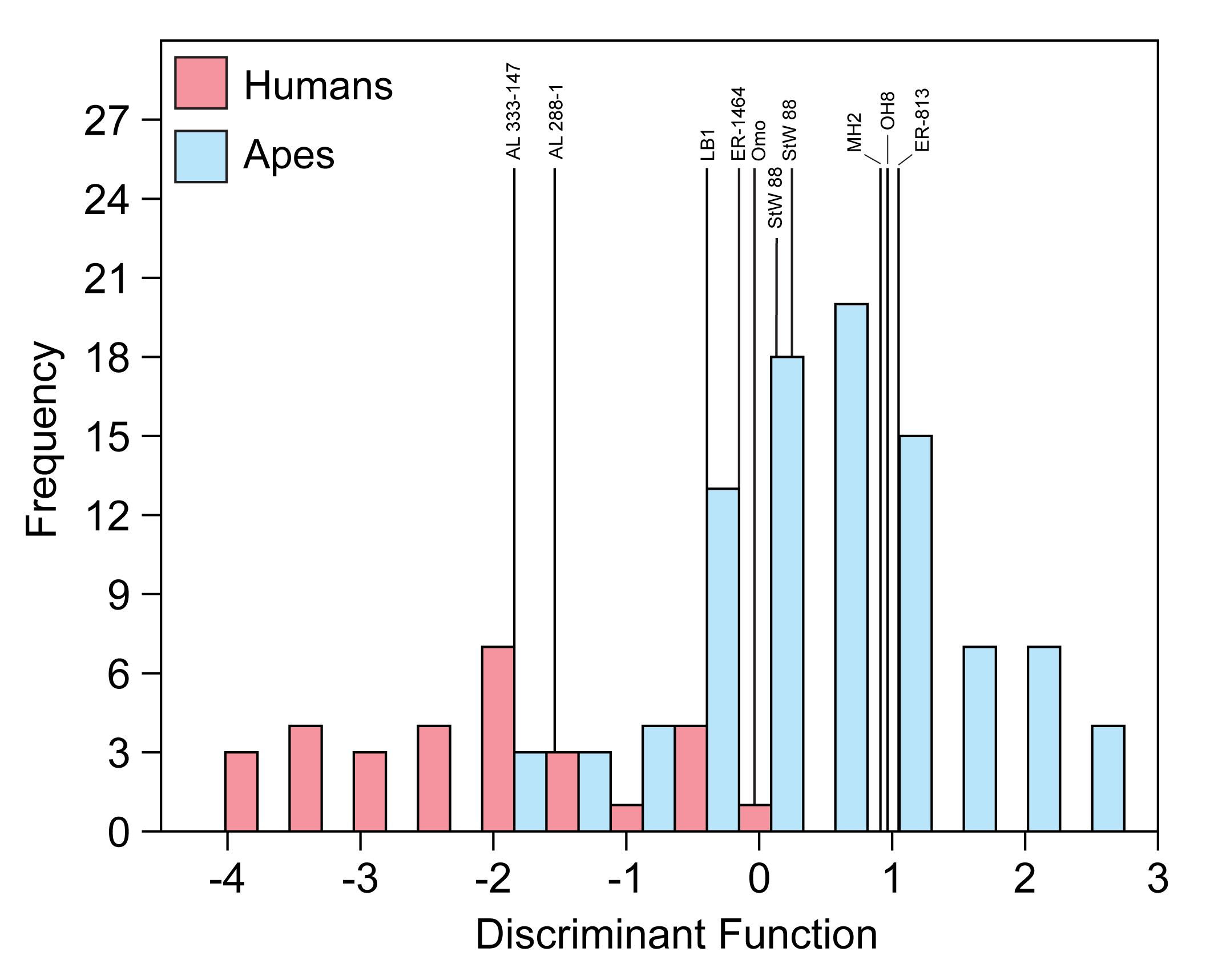


**Figure S5.** Discriminant function analysis using 3 talar variables (troch-nav, troch-cala, cala-nav), which shows 100% of the total sample variance. 94% of apes and 83% of humans are classified correctly using both resubstitution and cross-validation methods. The multivariate analysis shows that the Omo 323-76-898 talus is more similar to apes in talar head sagittal plane orientation than to humans, whereas *A. afarensis* specimens are more similar to humans, despite their metric overlap in the talonavicular joint angle (variable troch-nav) with gorillas and humans. All hominins are classified as apes, except for *A. afarensis* specimens.
